# Rare Complications of Seizures in End-Stage Renal Disease: A Case Report

**DOI:** 10.7759/cureus.9980

**Published:** 2020-08-24

**Authors:** Mahmoud Haddad, Khalid Bashir, Ahmad Al Sukal, Bilal Albaroudi, Amr Elmoheen

**Affiliations:** 1 Emergency Medicine, Hamad Medical Corporation, Doha, QAT; 2 Medicine, Qatar University, Doha, QAT

**Keywords:** seizure disorder, renal osteodystrophy, esrd, bilateral femurs neck fracture, posterior shoulder dislocation, tempo mandibular joint dislocation

## Abstract

Patients with chronic kidney disease (CKD) that progresses to end-stage renal disease (ESRD) typically present with uremic symptoms. CKD causes renal osteodystrophy, which leads to disturbances in mineral and bone metabolism. Pathological bone fractures after seizures activity has been reported in literature.

In this study, we present what we consider the first case of combined bilateral femoral neck fractures, bilateral temporomandibular joint dislocations, and right shoulder anterior fracture dislocation in a patient who had a seizure activity due to electrolyte imbalance resulting from ESRD. The patient is a 36-year-old man with CKD that progressed to ESRD.

Joint dislocations and bone fractures are rare complications of seizures activity. Diagnosis is usually delayed due to the low prevalence of these complications after seizures. Clinicians should always bear in mind that ESRD places patients at high risk of these rare complications.

## Introduction

Patients with chronic kidney disease (CKD) that progresses to end-stage renal disease (ESRD) present with many signs and symptoms, including fluid overload, electrolyte imbalance, metabolic acidosis, hypertension, anemia, and mineral and bone disorders [1]. Renal osteodystrophy is a term that denotes disturbances in mineral and bone metabolism due to CKD. It is associated with significant morbidity [2]. Pathological bone fractures after seizure activity have been reported in literature [3-16].

We herein present what we consider the first case of combined bilateral femoral neck fractures, bilateral temporomandibular joint dislocations, and right shoulder anterior fracture dislocation in a patient who had seizures due to electrolyte imbalance resulting from ESRD.

## Case presentation

**Figure 1:**
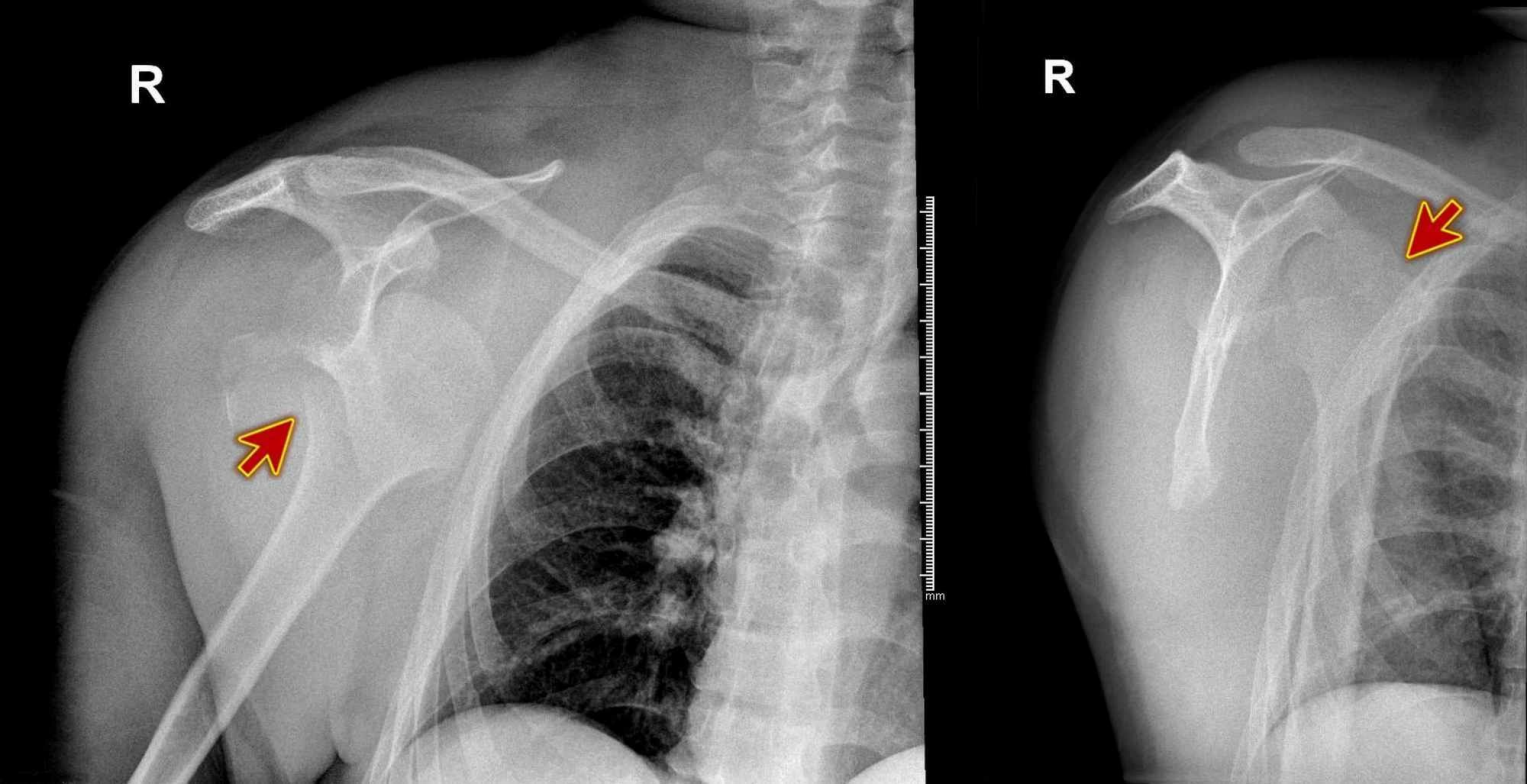
X-ray of the right shoulder showing anterior dislocation and fracture of the humeral head.

**Figure 2:**
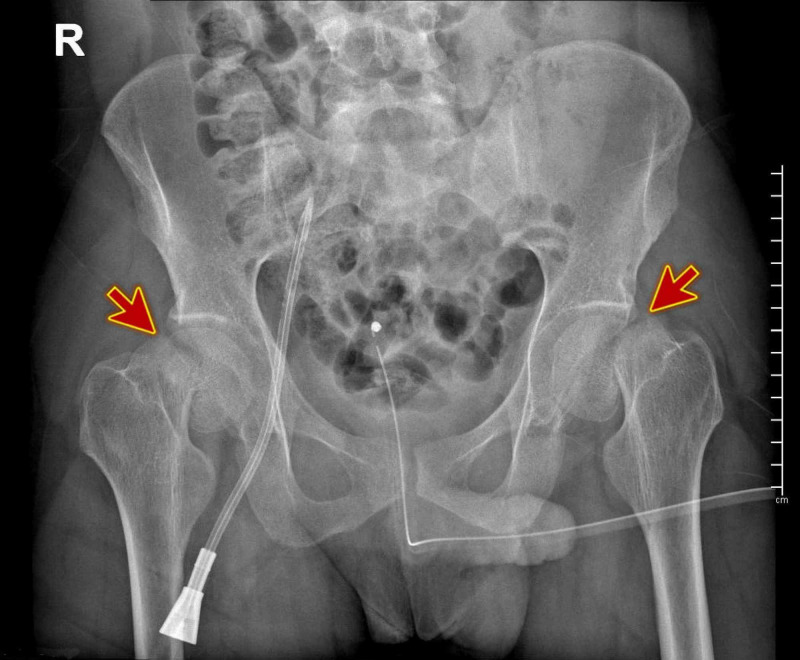
Pelvic X-ray showing bilateral displaced femoral neck fractures.

**Figure 3:**
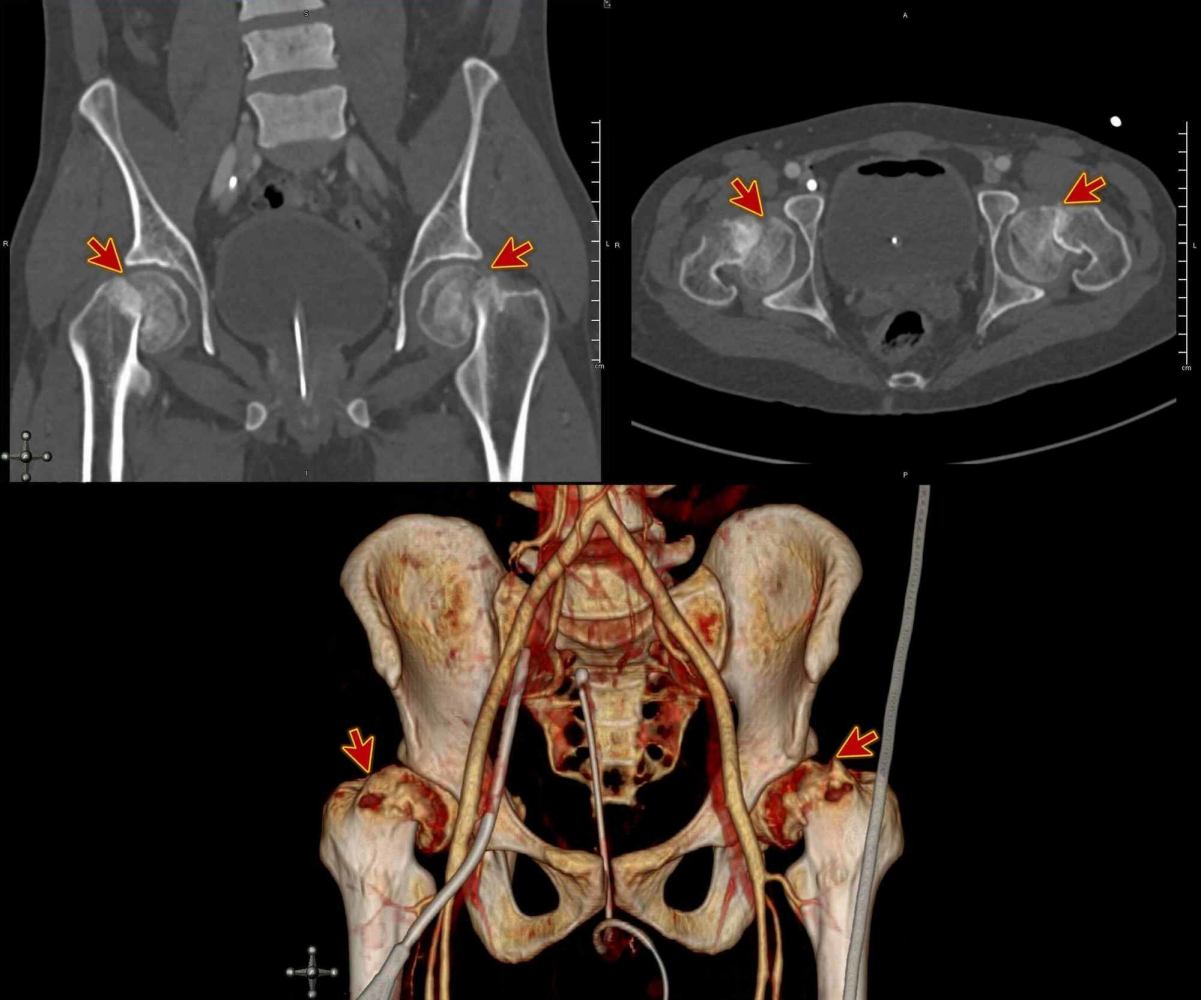
Pelvic CT showing bilateral displaced femoral neck fractures.

A 36-year-old Indian man with a medical history significant for CKD and hypertension presented to the ED after a witnessed episode of new-onset seizure at home that was followed by another episode of generalized tonic-clonic seizure in the ED. The seizures each lasted for less than two minutes and were followed by a postictal state. He complained of generalized body weakness, loss of appetite, nausea, and vomiting for the past three days, and there was no history of a fall or trauma. Patient assessment revealed a heart rate of 96 bpm and a blood pressure of 150/111 mmHg. He was alert and oriented with a Glasgow Coma Scale score of 15. However, we observed that the patient had a locked jaw and was unable to close his mouth. The patient also had tenderness of the right shoulder, and the range of motion of both his hips was reduced. His creatinine and urea levels were significantly elevated, and venous blood gas analysis revealed high anion-gap metabolic acidosis with hyponatremia, hypokalemia, and severe hypocalcemia. Therefore, he was moved to the resuscitation area of the ED for urgent hemodialysis (HD). The patient was consciously sedated, and his jaw was reduced using the traditional technique. Two attempts at closed shoulder reduction proved unsuccessful (Figure 1). A central venous catheter was inserted into the right femoral vein, electrolytes were administered, and HD was started. The patient still had a complaint of bilateral hip pain; therefore, pelvic X-ray was performed. To our surprise, the X-ray showed bilateral femoral neck fractures (Figure 2). Given the unexpected significant finding of bone injuries, we requested pan-CT to rule out other injuries. The pan-CT showed a single rib fracture and bilateral displaced femoral neck fractures (Figure 3) but was otherwise unremarkable. 

.

 

**Figure 4:**
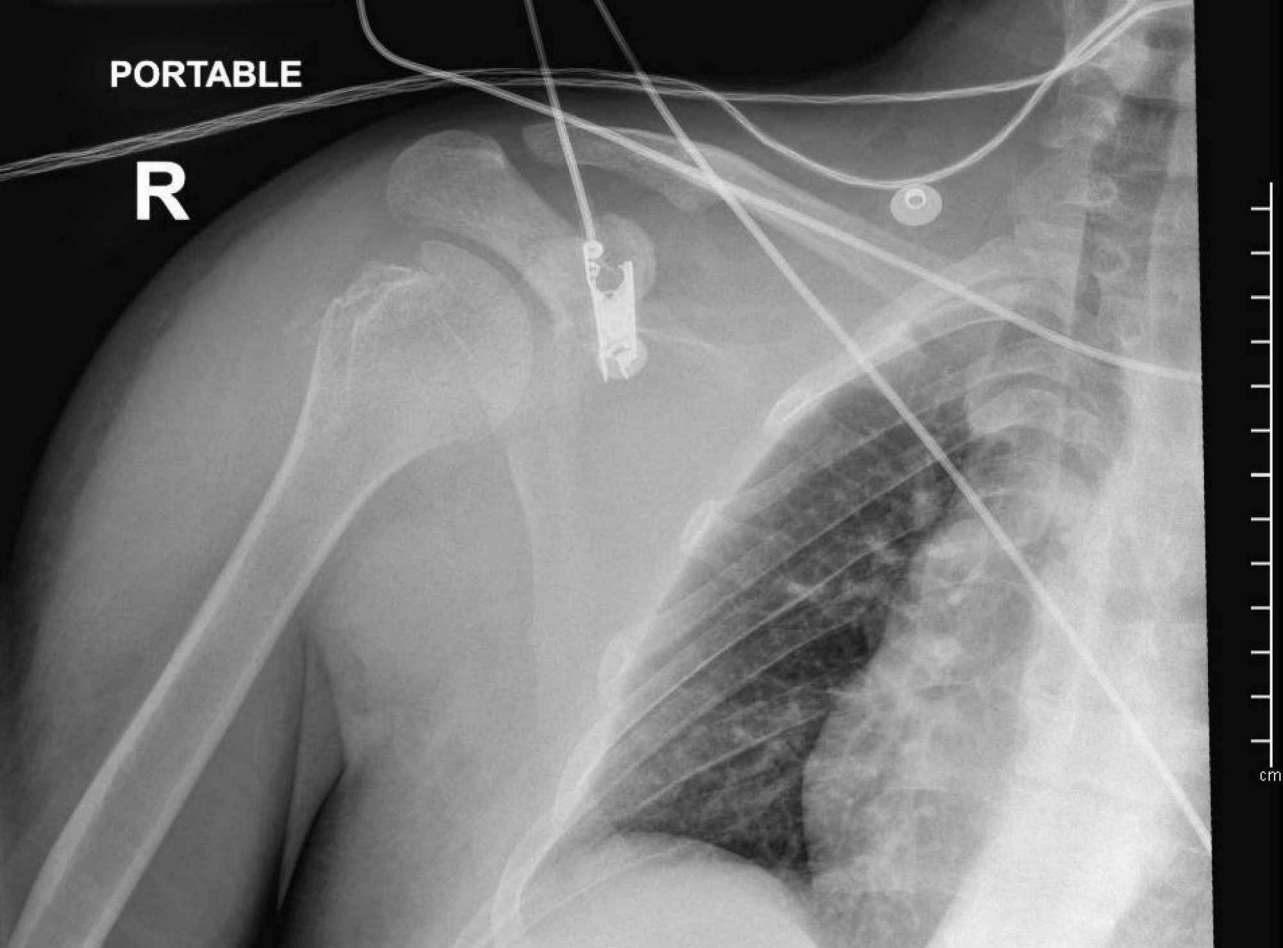
X-ray of the right shoulder after reduction.

**Figure 5:**
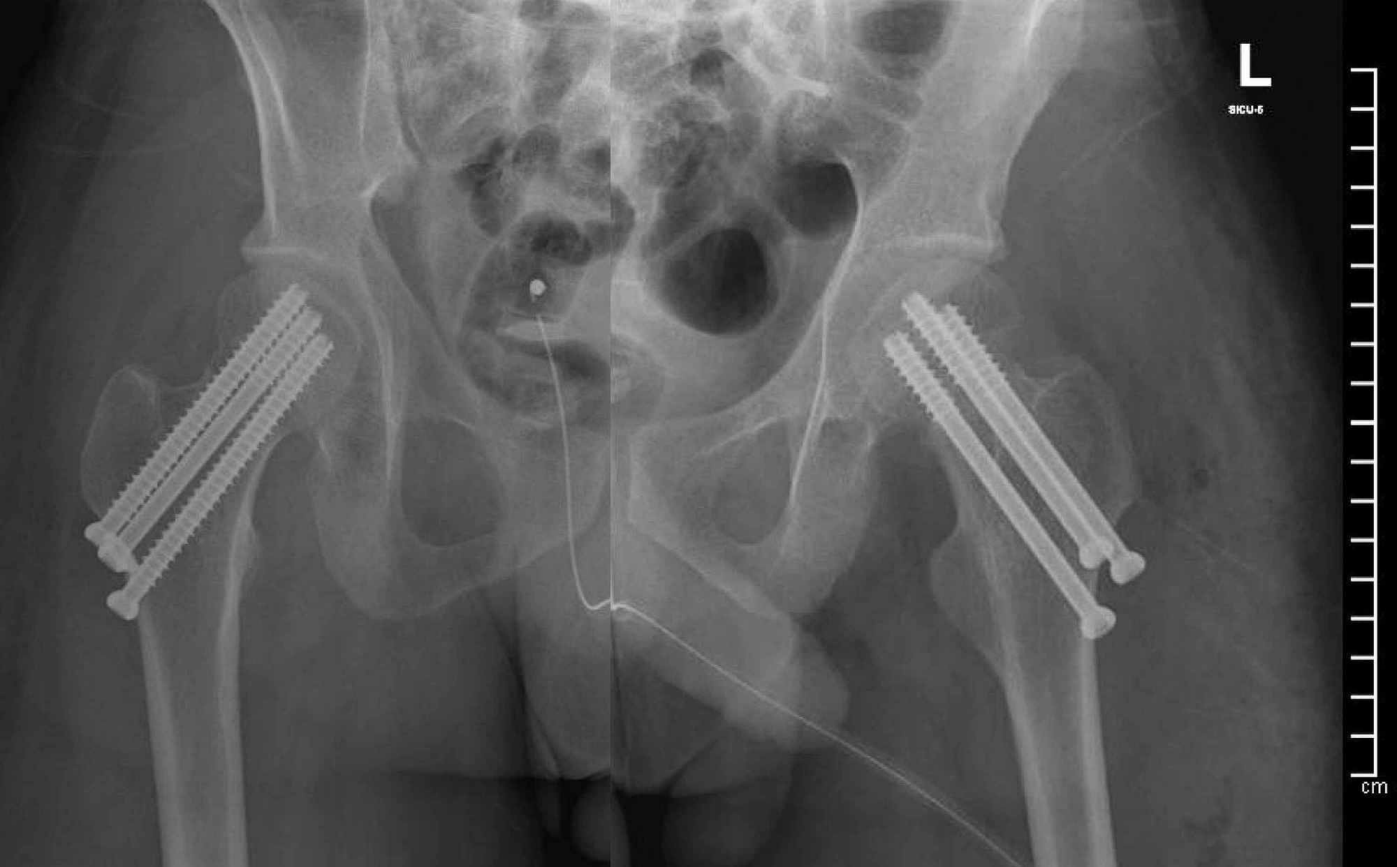
Pelvic X-ray after surgical fixation of femoral neck fractures.

The patient was admitted to the surgical ICU, and the right shoulder dislocation was reduced under conscious sedation (Figure 4). He underwent Permcath insertion, and HD was continued. Closed reduction and cancellous screw fixation of the bilateral femoral neck fractures was performed the following day (Figure 5). 

## Discussion

Chronic kidney disease-mineral and bone disorder (CKD-MBD) is well described in literature and mostly develops in stage III CKD. It is a systemic disorder of mineral and bone metabolism due to CKD and is characterized by one or a combination of abnormalities of calcium metabolism, phosphorus metabolism, parathyroid hormone (PTH) metabolism, vitamin D metabolism, bone mineralization, bone strength, bone linear growth, bone turnover disease, vascular calcification, and soft tissue calcification [17]. Typically, due to renal osteodystrophy, patients with CKD-MBD have low calcium levels and high susceptibility to bone fractures after minor trauma [17].

A study by Hughes-Austin et al. conducted on CKD patients, reported the biomarkers of bone turnover that help with the identification of patients’ subsets at high risk of bone fractures. All patients in the study underwent bone biopsy and histomorphometry. The author reported that patients whose bone biopsy revealed low bone turnover (low fibroblast growth factor 23, low PTH, and high alpha-klotho levels) have an eightfold risk of fractures [18].

First-time seizure is a serious event that requires proper assessment and management. It has various etiologies, including life-threatening provoking factors. A first-time seizure can also be an unprovoked seizure, which is usually due to the onset of epileptic disease. Accidents, injuries, and death associated with first-time seizures are under-reported [19]. Seizures are associated with injuries, and the majority of these injuries are minor injuries, such as head concussion and head laceration; however, major injuries, such as joint dislocations and bone fractures, may occur but are rare [20]. Joint dislocations and bone fractures following seizures are results of strong repetitive muscular contractions [11].

Posterior and anterior shoulder dislocations are a common complication following seizure activity. It is well described in literature [3-6]. Bilateral temporomandibular joint dislocations and bilateral femoral neck fractures are rare complications of seizures [7-16].

In many cases, the diagnosis of associated joint dislocations or bone fractures is delayed due to low clinical suspicion [3-16]. In the case of our patient, the diagnosis of bilateral femoral neck fractures was somewhat delayed due to other common complications and life-threatening electrolyte derangements resulting from ESRD and urgent HD initiation, which masked the femoral fractures. Our patient’s case is unique as no previous case studies have reported injuries, i.e. fractures and fracture dislocations at three joints as seizure complications.

The physiology, characteristics, and complication susceptibility of patients should always be considered as in the case of our patient, who had renal osteodystrophy, and susceptibility to fractures.

## Conclusions

Patients with ESRD usually develop renal osteodystrophy, which can predispose them to low bone density and put them at high risk of bone fractures following minor traumas. Joint dislocation and bone fracture are rare complications of seizure activity. Diagnosis of these complications in seizure patients is usually delayed due to the low prevalence of the complications. Clinicians should always consider patient physiology and the risk factors that can predispose patients to less common complications.
